# Secondary aerosol formation during the dark oxidation of residential biomass burning emissions†

**DOI:** 10.1039/d2ea00031h

**Published:** 2022-08-23

**Authors:** John K. Kodros, Christos Kaltsonoudis, Marco Paglione, Kalliopi Florou, Spiro Jorga, Christina Vasilakopoulou, Manuela Cirtog, Mathieu Cazaunau, Bénédicte Picquet-Varrault, Athanasios Nenes, Spyros N. Pandis

**Affiliations:** Institute of Chemical Engineering Sciences, ICE-HT Patras 26504 Greece athanasios.nenes@epfl.ch spyros@chemeng.upatras.gr; Institute of Atmospheric Sciences and Climate, Italian National Research Council Bologna 40129 Italy; Department of Chemical Engineering, Carnegie Mellon University Pittsburgh 15213 USA; Univ Paris Est Creteil and Université Paris Cité, CNRS, LISA F-94010 Créteil France; LISA, UMR CNRS 7583, Université Paris-Est Créteil, Université de Paris, Institut Pierre Simon Laplace (IPSL) Créteil France; School of Architecture, Civil and Environmental Engineering, Swiss Federal Institute of Technology Lausanne Lausanne 1015 Switzerland

## Abstract

Particulate matter from biomass burning emissions affects air quality, ecosystems and climate; however, quantifying these effects requires that the connection between primary emissions and secondary aerosol production is firmly established. We performed atmospheric simulation chamber experiments on the chemical oxidation of residential biomass burning emissions under dark conditions. Biomass burning organic aerosol was found to age under dark conditions, with its oxygen-to-carbon ratio increasing by 7–34% and producing 1–38 μg m^−3^ of secondary organic aerosol (5–80% increase over the fresh organic aerosol) after 30 min of exposure to NO_3_ radicals in the chamber (corresponding to 1–3 h of exposure to typical nighttime NO_3_ radical concentrations in an urban environment). The average mass concentration of SOA formed under dark-oxidation conditions was comparable to the mass concentration formed after 3 h (equivalent to 7–10 h of ambient exposure) under ultraviolet lights (6 μg m^−3^ or a 47% increase over the emitted organic aerosol concentration). The dark-aging experiments showed a substantial increase in secondary nitrate aerosol (0.12–3.8 μg m^−3^), 46–100% of which is in the form of organic nitrates. The biomass burning aerosol pH remained practically constant at 2.8 throughout the experiment. This value promotes inorganic nitrate partitioning to the particulate phase, potentially contributing to the buildup of nitrate aerosol in the boundary layer and enhancing long-range transport. These results suggest that oxidation through reactions with the NO_3_ radical is an additional secondary aerosol formation pathway in biomass burning emission plumes that should be accounted for in atmospheric chemical-transport models.

## Abstract 2

Biomass burning emissions age rapidly in the dark due to oxidation reactions with nitrate radicals.

## Graphical abstract

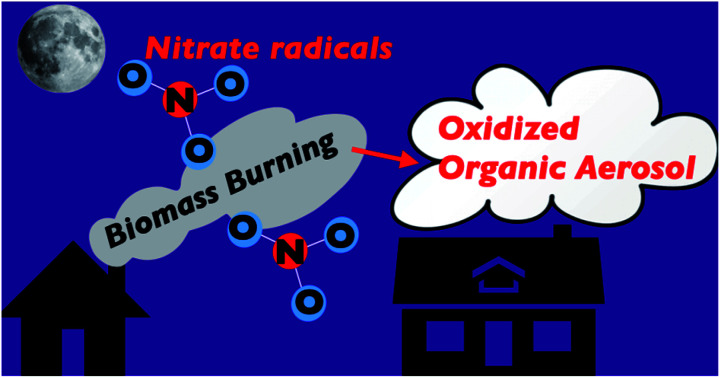

**Environmental significance**

Understanding the pathways for chemical aging of biomass burning emissions is essential to identifying and quantifying the impact of this source on air quality and climate; however, the role of chemical oxidation occurring in the absence of sunlight is not well understood. We present evidence of rapid dark chemical oxidation of biomass burning emissions and secondary aerosol production under laboratory conditions. This overnight production of secondary aerosol may contribute to aerosol burdens in winter urban environments. Thermodynamic modeling suggests that the pH of biomass burning remains at a level that can further promote inorganic aerosol production. This dark oxidation is not included in chemical transport models thus under-representing the link between biomass burning and its impact on health and climate.

## 1 Introduction

Biomass burning (BB), both in the form of open fires (including wildfires) and residential burning, is an increasingly important source of ambient particulate matter (PM) air pollution, affecting both climate and human health.^1–5^ BB emits substantial amounts of primary organic aerosol (POA), black carbon (BC), and brown carbon (BrC) directly into the particle phase.^2,6–9^ These primary emissions are a major contributor to global organic aerosol (OA) and absorbing aerosol burdens.^2^ BB is also an important contributor to the oxidative potential of PM, which is currently thought to be one of the causes of adverse health outcomes from PM inhalation.^10,11^

BB emits semi (SVOCs), intermediate (IVOCs), and volatile organic compounds (VOCs) directly into the atmosphere.^12^ Moreover, as the plume dilutes, POA can evaporate and further contribute to SVOC and IVOC availability.^13,14^ These organic vapors may react with oxidants in the atmosphere (*e.g.*, OH, O_3_, and NO_3_) to form lower-volatility compounds, which may then condense to the particle phase forming secondary organic aerosol (SOA). These chemical and physical transformations within the BB plume may form SOA on a time scale of hours to days further impacting climate and air quality.^14–17^ This SOA is often chemically different to the POA emitted directly from the BB source,^18,19^ complicating the link between BB emissions and net PM concentrations in the days following emission. In order to fully quantify the impacts of BB plumes on human health and climate, it is necessary to account for both the corresponding primary and secondary PM.

Previous research has mainly focused on the oxidation of BB plumes in the presence of sunlight (*i.e.*, *via* the OH radical).^18,20–22^ While the conditions that lead to net OA formation (the increase in SOA outweighs the decrease in POA due to dilution and evaporation) are not entirely understood, it is clear that BB plumes can undergo extensive chemical processing resulting in SOA production through oxidation with the OH radical. Hennigan *et al.*^23^ performed laboratory smog chamber experiments on BB emissions from a variety of fuels and reported a wide range of outcomes ranging from a net OA increase to a net decrease across all experiments. Hodshire *et al.*^22^ reported that laboratory experiments often showed a larger net increase in OA compared to field studies; however, both laboratory and field studies show extensive production of chemically aged aerosol, suggesting substantial SOA formation even if net OA remains roughly constant.

The established understanding of the daytime formation mechanism for SOA production has led to increasing complexity in atmospheric models, which are better able to reproduce observations under conditions of adequate sunlight.^24–27^ Fountoukis *et al.*^27^ found that oxidized OA (OOA) is fairly well represented in Paris in the summer; however, the model under-predicted OOA concentrations in the winter, suggesting a possible additional formation pathway for SOA under conditions of low-photochemical activity. One such proposed secondary mechanism for SOA production in BB plumes is *via* reactions with the NO_3_ radical.^28^

The NO_3_ radical is formed primarily through the reaction of NO_2_ and O_3_, and is rapidly photolyzed during the day.^29,30^ Thus, oxidation through reactions with the NO_3_ radical may only take place during periods of low photochemical activity (such as at night or in winter). Measurements of nighttime NO_3_ radical concentrations display a large degree of spatiotemporal variability. Two-year measurements of NO_3_ in an urban site in Jerusalem indicated an average nighttime concentration of 27 ± 43 ppt, with measured levels during 21 nights exceeding 220 ppt.^31,32^ The NO_3_ radical concentration has also been shown to exhibit a strong vertical gradient.^33^ Brown *et al.*^34^ found that on average, NO_3_ levels in a New England area increase with altitude reaching a maximum within the first 500 meters above the surface (with concentrations ranging from 0–90 ppt) and decrease at higher elevations. Urban and rural surface-layer measurements show average nighttime NO_3_ radical concentrations in the range of 10–40 ppt: Wang *et al.*^35^ reported 16 ± 9 ppt in Shanghai, China; Li *et al.*^36^ measured 22 ± 2 ppt in Guangzhou, China; McLaren *et al.*^37^ found median overnight concentrations of 10 ppt near Vancouver, Canada. Conversely, concentrations in the range of 3–10 ppt have been reported in remote sites.^38–40^ The chemical transport simulations of Khan *et al.*^29^ suggest that annual-mean nighttime NO_3_ radical concentrations ranged between 10–25 ppt across the northeastern US.

Studies to date have established that the NO_3_ radical is a very efficient oxidant of many biogenic VOC emissions, particularly during periods of transport and mixing with air with high NO_*x*_ concentrations.^30,41–45^ In particular, the NO_3_ radical has been shown to be very reactive toward unsaturated VOCs. Recently, there has been increasing interest in exploring the extent of nighttime chemical processing of BB emission plumes. Tiitta *et al.*^46^ and Hartikainen *et al.*^47^ reported substantial SOA production (factor of 2 increases over the emitted POA concentration) in laboratory experiments under dark conditions, with dominant gas-phase reactions taking place between the NO_3_ radical and phenolic and furanoic compounds. Decker *et al.*^28^ initialized a chemical box model with aircraft observations and predicted substantial overnight oxidation of BB VOCs largely through reactions with NO_3_. Li *et al.*^36^ found substantial absorption enhancement of brown carbon after NO_3_ radicals were allowed to react with tar aerosols from BB in a flow reactor. Kodros *et al.*^16^ argued that the rapid and extensive chemical processing of BB plumes in the dark observed in laboratory experiments produces SOA that is chemically similar to ambient observations of oxidized and secondary OA factors, thus suggesting dark oxidation of BB plumes may be an additional formation pathway of OOA not included in most models.

Dark oxidation of BB plumes *via* the NO_3_ radical may also lead to secondary inorganic and organic nitrate aerosol formation.^16,28,46^ Kiendler-Scharr *et al.*^48^ found substantial organic nitrate mass concentrations in PM in urban and rural areas and estimated that the dominant formation pathway involves reactions with the NO_3_ radical. Similarly, Rollins *et al.*^49^ reported organic nitrate aerosol production as a result of nocturnal NO_3_ radical chemistry in Bakersfield, California. The contribution of residential BB specifically to organic and inorganic nitrate aerosol through dark oxidation in urban areas is not well understood.

Biomass burning aerosol exhibits substantial hygroscopicity.^50^ Biomass burning emits considerable amounts of NH_3_ and NO_*x*_, that in turn can transform into inorganic nitrate aerosol. The paucity of sulfate and the presence of potassium and other non-volatile cations also imply that the aerosol generated during biomass burning is much less acidic than other types of combustion aerosol (*e.g.*, from fossil fuels). Observations^51,52^ and model studies to date^53^ suggest that ambient aerosol dominated by biomass burning emissions tends to exhibit elevated levels of pH compared to other aerosol types. This in turn carries important implications for the susceptibility of the aerosol to NH_3_ and HNO_3_ levels, as well as the tendency of inorganic nitrate aerosol to accumulate in the boundary layer during BB events and the resulting nitrogen being able to transport over long distances before deposition.^54,55^ It is unclear if the elevated pH levels are an “inherent” property of BB aerosol, and if it is driven primarily by the semi-volatile inorganic components (NH_3_, HNO_3_) or the non-volatile cations that may be present (primarily K^+^). Finally, the importance of the organic water in controlling aerosol pH levels also needs to be determined.

In this work we investigate if BB emissions can age rapidly in the dark and form substantial concentrations of secondary organic and inorganic aerosol. This dark oxidation is not included in most atmospheric chemical-transport models, suggesting an under-representation of the link between BB emissions and secondary aerosol. In Section 2, we detail the environmental smog chamber experimental procedure and instrumentation. In Section 3, we discuss secondary aerosol formation and mass spectra evolution for a typical experiment (Section 3.1), discuss the range of results across all experiments (Section 3.2), calculate aerosol acidity (Section 3.3), and finally estimate the atmospheric relevant timescales of the major processes (Section 3.4). In Section 4, we discuss our conclusions and study limitations.

## 2 Methods

### 2.1 Experimental facility and instrumentation

Experiments on the dark chemical processing of BB emissions took place at the Foundation for Research and Technology-Hellas (FORTH) atmospheric simulation chamber and combustion chamber facilities at the Center for the Study of Air Quality and Climate Change. This facility consists of a 30 m^3^ temperature and light-controlled room capable of sustaining variable sized reactors. In this study, all experiments took place in a 10 m^3^ Teflon reactor. Combustion of biomass took place in the combustion facility located in a separate room directly beneath the chamber. BB emissions were transferred into the chamber through a dilution system.

In the chamber, a suite of online instrumentation measured the concentrations of particle- and gas-phase species. Non-refractory PM_1_ aerosol was monitored by a high-resolution time-of-flight aerosol mass spectrometer (HR-ToF-AMS, Aerodyne Research Inc.) working in V mode. A scanning mobility particle sizer (SMPS; classifier model 3080, DMA model 3081, CPC model 3787, TSI) measured the aerosol number size distribution. In these experiments, the sheath flow rate was 3 L min^−1^ and the aerosol sample flow rate was 0.6 L min^−1^, allowing for a diameter size range of 14 to 790 nm. Black carbon was monitored using a multiple-angle absorption photometer (MAAP, Thermo Scientific Inc.) and a single particle soot photometer (SP2, Droplet Measurement Technology). A proton-transfer-reaction mass spectrometer (PTR-MS, Ionicon Analytik) measured VOCs. Inorganic gas-phase species were measured using a series of gas monitors: nitrogen oxides (NO and NO_2_, Teledyne model T201), ozone (O_3_, Teledyne model 400E), carbon monoxide (CO, Teledyne model 300E), and carbon dioxide (CO_2_, Teledyne model T360). In a subset of experiments, NO_3_ radical concentrations were measured by incoherent broad-band cavity-enhanced absorption spectroscopy (IBB-CEAS) as described by Fouqueau *et al.*^56^ Gas-phase concentrations of NH_3_ were measured using a photoacoustic monitor (LSE, model NH_3_-1700).

### 2.2 Experimental procedure

All eight dark-aging experiments (experiments 1–8; Table 1) followed the same general procedure. First, the BB emissions were injected into the chamber through the dilution system (with a dilution rate of approximately 10-to-1). In all experiments, a small amount (approximately 40–70 ppb) of d9-butanol was also injected to determine the OH concentrations. The fresh emissions remained in the chamber under dark conditions for approximately 2 hours to allow sufficient time for mixing and characterization. During this period, some chemical processing may occur through reactions among the primary pollutants and other compounds already present in the mixture (*e.g.* ozone). To initiate oxidation by NO_3_ radicals, which we defined as time zero in all experiments, we injected a variable concentration of NO_2_ and O_3_ (precursors to the NO_3_ radical). We then allowed at least 3 hours for chemical processing and characterization of the aged aerosol and vapors. In a subset of experiments, we injected ammonium sulfate immediately following the chemical processing period in order to quantify the size-resolved loss rates of particles to the chamber walls. We also performed 2 reference experiments (experiments 9–10). In experiment 9, we initiated oxidation (at time zero) through turning on ultraviolet (UV) lights to simulate daytime (OH radical) oxidation. In experiment 10, we left the BB emissions in the chamber under dark conditions for several hours without injection of NO_2_ and O_3_.

**Table 1:** Aerosol emissions and properties for each biomass burning experiment along with experimental conditions and injected mixing ratios of O_3_ and NO_2_

Exp.	Biomass burning emissions					Experimental conditions				
	OA [μg m^−3^]	Inorganic nitrate [μg m^−3^]	Organic nitrate [μg m^−3^]	BC [μg m^−3^]	O : C	Lights	RH [%]	*T* [K]	O_3_ [ppb]	NO_2_ [ppb]
1	14	0.2	0.2	7.9	0.3	Dark	9	27	123	93
2	14	0.1	0.1	3.8	0.4	Dark	9	21	44	43
3	83	0.7	0.8	109	0.3	Dark	10	25	228	99
4	39	0.4	0.2	0.8	0.4	Dark	58	27	91	57
5	33	0.7	0.4	7.0	0.4	Dark	58	28	52	36
6	9	0.7	0.4	36	0.4	Dark	48	27	143	70
7	47	0.8	0.5	4.5	0.4	Dark	45	27	173	97
8	30	0.8	0.7	0.1	0.4	Dark	60	26	118	103
9	13	0.2	0.2	6	0.4	UV	15	19	—	—
10	45	0.5	0.4	8.4	0.4	Dark	8	21	(23)^a^	(7)^a^

a In experiment 10, no additional NO_2_ or O_3_ was injected. The levels reported here represent emissions directly from combustion as a comparison.

Initial concentrations of biomass burning aerosol, NO_2_ and O_3_, as well as relative humidity are presented for all experiments in Table 1. Initial OA concentrations measured by the AMS ranged from 9–83 μg m^−3^ across all experiments. To simulate oxidation in dark conditions in urban areas, we injected between 36–103 ppb of NO_2_ and 44–228 ppb of O_3_ for each experiment. In all experiments, NO_2_ was injected prior to the injection of O_3_ to prevent reaction of ozone with organic compounds. The relatively high concentrations of NO_2_ and O_3_ are intended to accelerate oxidation that may take place over night. Section 3.4 discusses the corresponding atmospheric timescales which are longer than those of our experiments. Finally, to test the sensitivity of dark oxidation to relative humidity and possible contribution of aqueous-phase oxidation to chemical processing, we varied the relative humidity in the chamber ranging from dry conditions (about 10% relative humidity) to moderate levels of around 60%. The temperature of the experiments ranged from 19–28 °C, values that are in general higher than those typically expected for winter nighttime conditions. We expect these higher temperatures to favor NO_3_ radical production. This issue will also be addressed in Section 3.4.

The combustion of olive tree wood in a residential wood stove was the BB source of our experiments. Both the wood and stove were purchased locally and represent typical residential heating devices and fuels used in Greece. Previous studies have suggested that combustion conditions such as flaming phase or combustion temperature may play a role in explaining the variability of the extent of oxidation by altering the emission profile of particle- and gas-phase organic compounds. In this study, we do not systematically test the sensitivity of dark oxidation to combustion conditions. In all experiments, we sampled under flaming conditions, roughly representative of expected conditions in a residential wood stove, approximately 15–30 minutes after ignition. We note that differences in combustion conditions may play some role in the extent of oxidation through differences in emission profiles.

### 2.3 Data analysis

The HR-ToF-AMS measurements were analyzed using the AMS software toolkits SeQUential Igor data RetRiEvaL (SQUIRREL) v1.57 and Peak Integration by Key Analysis (PIKA) v1.16. Elemental ratios (*e.g.*, O : C) were calculated following the method of Canagaratna *et al.*^57^ The AMS collection efficiency was calculated following the method of Kostenidou *et al.*^58^ The total nitrate aerosol measured by the AMS was apportioned into organic and inorganic nitrate following the methods discussed in Farmer *et al.*^59^ and Kiendler-Scharr *et al.*^48^ While we recognize the uncertainty in this method, we feel its use is acceptable for the purposes of this study.

The measured size-dependent particle wall-loss rates measured during the characterization period after an experiment were used to correct the AMS mass concentrations following Wang *et al.*^60^ The relative standard deviation of the measured wall loss rate constants across experiments was 35%. In experiments where a wall-loss characterization period did not take place directly following oxidation, the average size-resolved particle wall loss rate profile was used to correct for particle wall loss.

The secondary aerosol mass concentration at time *t* is defined as the wall-loss corrected mass concentration minus the average wall-loss corrected mass concentration during the hour before the start of oxidation. The enhancement ratio (ER) of a parameter is defined as the parameter measured at time *t* divided by the averaged parameter before the start of oxidation. It has been calculated for the wall-loss corrected mass concentration and for the O : C ratio.

To separate the total BB OA (bbOA) AMS spectrum into a primary (bbPOA) and a produced secondary OA (bbSOA) spectrum, we use a simplified mass-balance approach outlined in Jorga *et al.*^61^ In this method, we estimate the initial (*i.e.*, fresh) bbPOA assuming that its mass concentration is only affected by particle wall loss with a first-order loss rate with the wall loss rate calculated as discussed above, while the normalized bbPOA spectrum is assumed to be constant in time. The produced bbSOA spectrum is thus the difference between the measured OA spectrum and the estimated fresh bbPOA spectrum at time *t*. We note that this method assumes that particle wall loss and SOA formation are the dominant processes taking place in the chamber, and does not account for semi-volatile vapor wall loss or heterogeneous reactions.

We use the theta angle to quantify the differences between bbOA mass spectra.^62^ The theta angle treats two mass spectra as *n*-dimensional vectors (where *n* is the number of *m*/*z* values in the spectrum) and calculates the inner product between them. The theta angle is calculated by eqn (1) (following the discussion in Kostenidou *et al.*^62^):1
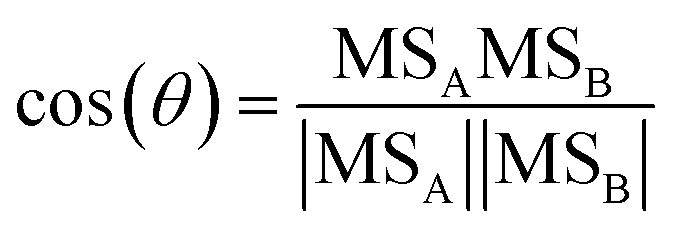
where MS_A_ and MS_B_ are the OA mass spectra expressed as vectors. In previous studies,^63^ subjective thresholds have been proposed to interpret the theta angle: an angle of 0–5° indicates a near-identical match between the spectra, 6–10° represents a high degree of similarity, 11–15° indicates some similarities between the spectra with notable differences, 16–30° suggests spectra from different sources with some shared similarities, while theta angles greater than 30° suggest notably different spectra. Here, we apply the theta angle in two main ways: to compare the spectra of different OA (for instance, the fresh bbPOA spectra and the produced bbSOA spectra) and the time evolution of the OA over the experiment (for instance, by comparing the spectra at time −1 hour to 3 hours). Kaltsonoudis *et al.*^63^ calculated theta angles ranging from 15–20° between fresh cooking OA generated in the laboratory and the same plume exposed to UV light for 3 hours in an environmental smog chamber.

In all experiments, OH concentrations are inferred through the consumption of d9-butanol injected at the start of the experiment.^64^ Here, we define one photochemical day as continued 24 h OH exposure at a concentration of 1.5 × 10^6^ molecule per cm^3^.^65,66^

### 2.4 Calculation of aerosol pH

To estimate the acidity of the BB emissions and the associated change after dark chemical aging, we simulate aerosol thermodynamic properties using ISORROPIA-Lite,^67^ which is based on the metastable routines of ISORROPIA II, version 2.3 (ref. 68) but expanded to include the effects of aerosol water uptake associated with the bbOA on the semi-volatile partitioning of the inorganics. Inputs to ISORROPIA-Lite include temperature, relative humidity, and the total (gas-plus particle-phase) concentrations of NH_3_, H_2_SO_4_, Na^+^, Ca^2+^, K^+^, Mg^2+^, HCl, and HNO_3_. In addition, water uptake from OA is calculated based on the OA mass concentration, density, and the hygroscopicity parameter, *κ*.^69^ Measurements from the AMS are used as inputs for the particle-phase species with the exception of the crustal elements and measurements of gas-phase NH_3_. Due to lack of measurements, we assume that the total HNO_3_ is equal to the measured particulate inorganic nitrate concentration (which is a reasonable assumption if the pH is high enough for most of the nitrate to partition to the particulate phase). We further assume that the concentrations of HCl, Na^+^, Ca^2+^, and Mg^2+^ are negligible. The lack of direct measurements of these species is a limitation of this analysis.

In our base set of calculations, we assume that concentrations of potassium (K^+^) are 0.15 times the concentration of particulate nitrate following Ryu *et al.*^70^ We set the OA hygroscopicity parameter equal to 0.15 and OA density to 1.4 g cm^−3^. The sensitivity of our results to the above assumptions has been quantified and is described in a subsequent section.

## 3 Results

### 3.1 Results of a typical dark oxidation experiment

**Fig. 1:**
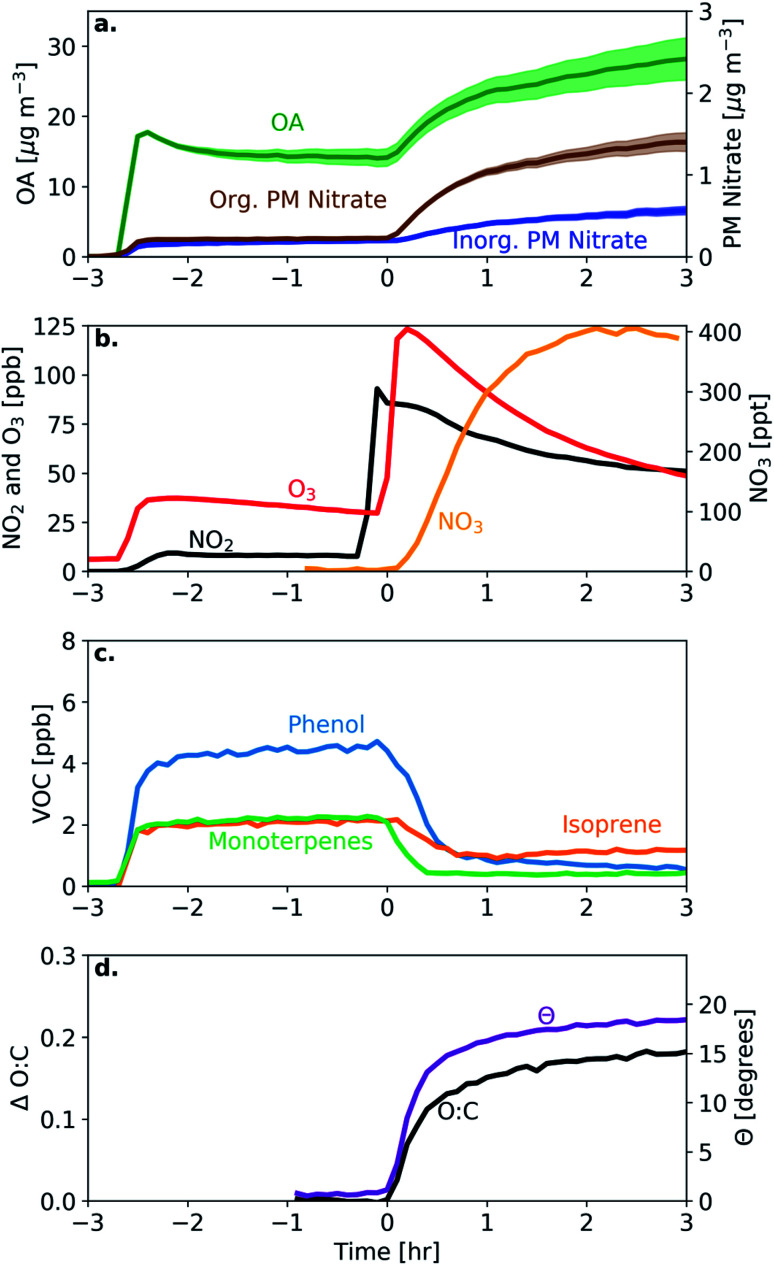
Measurements for a typical experiment (experiment 1 in Table 1) showing (a) wall-loss corrected bbOA, PM inorganic nitrate, and PM organic nitrate (shaded region represents one standard deviation around the wall-loss rate constant), (b) inorganic gas-phase species NO_2_, O_3_, and NO_3_ radical, (c) representative VOCs showing the largest decrease (phenol, isoprene, and monoterpenes), and (d) the change in the O : C ratio and theta angle.

In a typical experiment, such as experiment 1 (see Table 1), the BB emissions were transferred into the chamber at approximately −2.5 h (Fig. 1). The particles were composed of bbOA (14 μg m^−3^), 8 μg m^−3^ of BC and less than 1 μg m^−3^ each of nitrate, sulfate, ammonium, and chloride (Fig. 1a). The BB emissions also included 15 ppb of O_3_ and 11 ppb of NO_2_ (Fig. 1b). During the 2 h characterization period of the fresh emissions, the mass concentration of these particle- and gas-phase species shows only slight changes, with a decreasing trend of O_3_ (possibly due to reactions with NO and NO_2_ and losses to the chamber walls).

At time zero, dark oxidation was initiated through the injection of 93 ppb of NO_2_ and 123 ppb of O_3_, resulting in increasing NO_3_ radical concentrations (125 ppt after 30 min, Fig. 1b). Following this injection, rapid formation of bbSOA and PM organic nitrate is observed. In the first 30 min following the injection of O_3_, the bbOA mass increased by 5 μg m^−3^ (an enhancement of 37%), while the PM organic nitrate increased by 0.5 μg m^−3^ (and enhancement of almost 250%). About 40% of the secondary aerosol mass was formed in these first 30 min after the injection of O_3_. Also following injection, there was a corresponding decrease in VOC levels, the largest of which were phenol, isoprene, and monoterpenes (Fig. 1c).

To measure the extent of chemical processing of bbOA, we consider two metrics commonly associated with quantifying the extent of OA oxidation: the change in the O : C ratio and the evolution of the theta angle (defined in Section 2.3). In the hour prior to the initiation of oxidation both metrics remained roughly constant (the O : C ratio varied by less than 0.01 and the theta angle varied by less than 1°), suggesting little chemical changes of the fresh bbOA emissions (Fig. 1d). This suggests that on a broad level, BB emission plumes may not age rapidly in the dark without being exposed to sufficiently high concentrations of NO_2_ and O_3_ (that react to form the highly oxidizing NO_3_ radical). In the 30 min following the injection of NO_2_ and O_3_, the O : C ratio increases by 0.12 (34% increase), and the theta angle by 14°, indicating notable evolution of the bbOA spectrum while still maintaining some similarity to the fresh bbOA aerosol. The majority of the total increase in these metrics over the course of the experiment takes place in the first 30 min (65% and 74% of the total increase over the experiment in O : C and the theta angle, respectively).

**Fig. 2:**
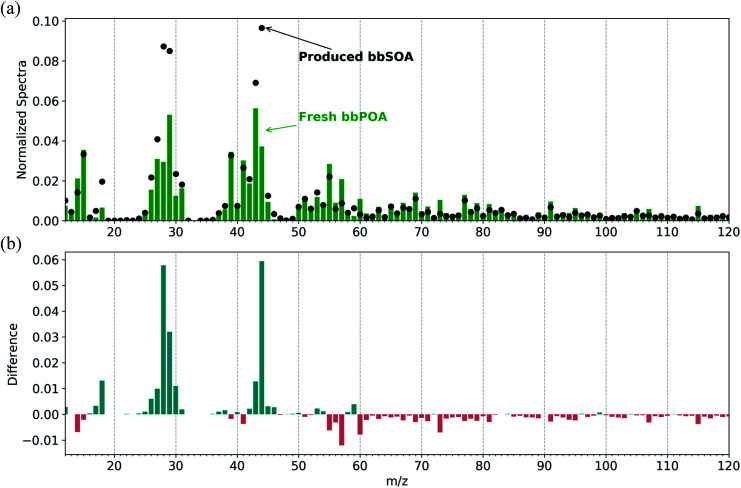
(a) The normalized fresh bbPOA (green bars) and produced bbSOA (black circles) factors and (b) the difference between them for the typical experiment (experiment 1). The blue bars indicate an increasing contribution while the red bars represent a decreasing contribution in OA at the given *m*/*z* for the produced bbSOA relative to the fresh bbPOA.

We separate the measured OA mass spectra into a fresh bbPOA and produced bbSOA spectrum (Fig. 2). Similar to previous studies on BB oxidation, the normalized produced bbSOA spectrum in these experiments shows enhancements at *m*/*z* 28 (CO^+^), 29 (CHO^+^), and 44 (CO_2_^+^), and decreases in *m*/*z* 60 (C_2_H_4_O_2_^+^), 57 (C_4_H_9_^+^), and 73 (C_3_H_5_O_2_^+^), relative to the normalized fresh bbPOA spectrum (Fig. 2). Both the fresh bbPOA and the produced bbSOA display prominent peaks commonly associated with combustion at *m*/*z* 69 (C_4_H_5_O^+^) and 91 (C_7_H_7_^+^). The theta angle between the fresh and aged factor is 30°, indicating substantial dissimilarity between the two spectra.

To estimate the relative contribution of reactions with NO_3_, O_3_, and OH to the oxidation of BB emissions, we calculate the average lifetime of the VOCs with the largest decreasing trends: phenol, isoprene, and monoterpenes (assumed to be α-pinene for this calculation). As a simplification we use the concentrations of these VOCs in the fresh BB emissions and the average concentration of the oxidants in the first hour after the initiation of oxidation. For all three VOCs, the average lifetime for reactions with OH are greater than 9 hours (three times the length of the oxidation portion of the experiment), suggesting OH concentrations are too low (approximately 9.5 × 10^5^ molecule per cm^3^) to play an important role in the oxidation reactions in our experiments (Tables S2 and S3†). In contrast, the average lifetime against reactions with NO_3_ are all less than 1 h (0.9 h for phenol, 0.5 h for isoprene, and 0.08–0.16 h for α-pinene), indicating NO_3_ likely plays a dominant role in the oxidation of all three VOCs. Finally, reactions with O_3_ are likely only relevant for α-pinene (with an average lifetime of 1.9–2.9 h). While the reactions of phenol are most likely dominated by the NO_3_ radical, it is possible that O_3_ may react with products of this initial reaction. For instance, the reaction of phenol with NO_3_ produces a variety of products, one of which is the organic molecule, C_6_H_5_O. The average lifetime of C_6_H_5_O against reactions with O_3_ is less than 1 min (Table S3†). Additionally, O_3_ may react heterogeneously with organic compounds in the aerosol phase.

### 3.2 Results across all experiments

#### 3.2.1 Oxidation of bbOA and SOA formation

**Fig. 3:**
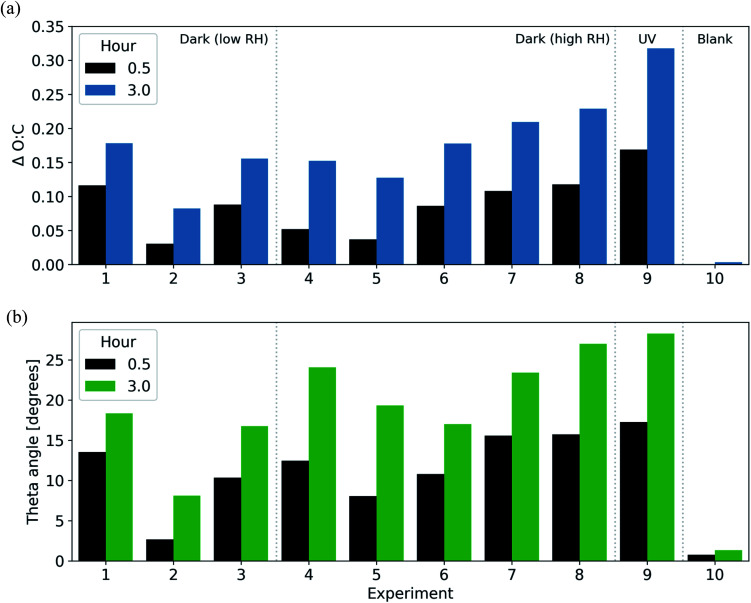
The change in (a) O : C ratio and (b) theta angle across all BB experiments. Experiments 1–3 were performed in dark and dry conditions, exp. 4–8 in dark and humid conditions, and exp. 9 and 10 are the reference experiments. The black bars represent the change in the first 0.5 h following the initiation of oxidation, while the green/black bars denote the change following 3 hours after the initiation of oxidation.

In all dark-aging experiments (experiments 1–8), the initial (prior to oxidation) O : C ratio ranged from 0.3–0.4. This ratio remained roughly constant over the 1–2 h prior to oxidation. Following the injection of NO_2_ and O_3_, all experiments show an increase in O : C ratio ranging from 0.03–0.12 (enhancement ratio of 1.07–1.34) after 30 min and 0.09–0.23 (enhancement of 1.2–1.58) after 3 h (Table 2). The experiments with the lowest concentration of injected NO_2_ and O_3_ (experiments 2 and 5) had the lowest increase in O : C ratio (0.03–0.04) after the first 30 min. Similarly, the theta angle increased in all dark-aging experiments by 3–16° in the first 30 min following the initiation of oxidation and by 8–27° after 3 h (Fig. 3). While experiments 2 and 5 had the lowest exposure to NO_2_ and O_3_ and corresponding lowest increase in O : C ratio, only experiment 2 had a similarly limited increase in theta angle (3° after 30 minutes) while the theta angle in experiment 5 increased by a modest 8° after the first 30 min. The reason for this discrepancy may be the effect of relative humidity. Experiment 2 was performed under dry (less than 10% RH) conditions, while experiment 5 was performed at an RH of 60%.

**Table 2:** Extent of oxidation in each BB experiment as represented by enhancement ratio (ER) and absolute difference in O : C ratio and the theta angle

Experiment	0.5 hours			3 hours		
	ΔO : C	O : C_ER_	Theta angle	ΔO : C	O : C_ER_	Theta angle
1	0.12	1.34	13.5	0.18	1.52	18.3
2	0.03	1.07	2.7	0.09	1.20	8.1
3	0.09	1.26	10.3	0.16	1.46	16.7
4	0.05	1.12	12.4	0.14	1.33	24.1
5	0.04	1.09	8.0	0.12	1.32	19.3
6	0.09	1.19	10.8	0.18	1.38	17.0
7	0.10	1.28	15.6	0.20	1.53	23.4
8	0.11	1.30	15.7	0.23	1.58	27.0
9	0.17	1.43	17.3	0.32	1.81	28.3
10	0.00	1.00	0.8	0.0	1.00	1.3

**Fig. 4:**
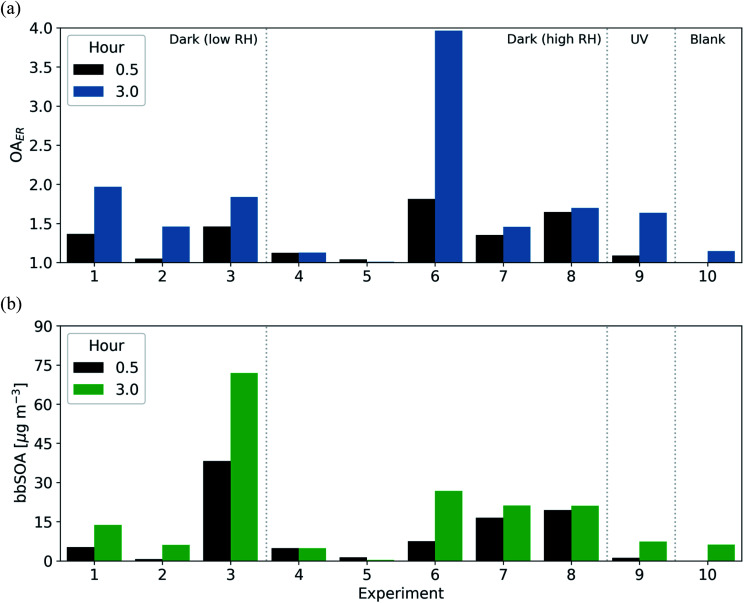
The (a) OA_ER_ and (b) bbSOA mass concentration after 30 minutes (black) and 3 hours (blue/green) for all dark oxidation and reference experiments.

All dark oxidation experiments showed production of SOA following injection of NO_2_ and O_3_ (Table 3 and Fig. 4). Wall-loss corrected SOA ranged from 0.7–38 μg m^−3^ (an OA ER of 1.05–1.81) in the 30 min following the initiation of oxidation. Between 11–53% of the total SOA was formed within these first 30 min; however, all experiments except for experiment 5 show a continued increasing trend in wall-loss corrected OA throughout the 3 h oxidation period. Similar to the O : C ratio, experiments with lower levels of NO_2_ and O_3_ (experiments 2 and 5) tended to form lower concentrations of SOA with OA enhancements in the range of 4–5% as compared to similar experiments with higher levels of NO_2_ and O_3_ with enhancements ranging from 24–81%.

**Table 3:** Extent of oxidation in each BB experiment as represented by enhancement ratio (ER) and absolute difference in bbSOA, organic-nitrates, and inorganic nitrate aerosol

Experiment	0.5 hours				3 hours			
	bbSOA [μg m^−3^]	OA_ER_	PM org. nitrate [μg m^−3^]	PM inorg. nitrate [μg m^−3^]	bbSOA [μg m^−3^]	OA_ER_	PM org. nitrate [μg m^−3^]	PM inorg. nitrate [μg m^−3^]
1	5.3	1.37	0.47	0.11	13.6	1.95	1.18	0.37
2	0.68	1.05	0.09	0.04	5.6	1.41	0.39	0.19
3	38.2	1.46	1.85	2.03	74	1.89	3.9	3.7
4	4.9	1.24	0.48	0.04	4.2	1.11	0.88	0.05
5	1.4	1.04	0.12	0.00	0.1	1.00	0.32	−0.1
6	7.5	1.81	0.30	0.47	26.6	3.9	1.3	1.5
7	16.5	1.35	1.49	0.41	21	1.44	2.17	0.53
8	19.4	1.64	1.17	0.45	21	1.70	1.70	0.26
9	1.16	1.09	0.05	0.02	6.1	1.47	0.08	0.03
10	0.00	1.00	0.08	0.06	3.9	1.1	0.20	0.20

We compare these dark-oxidation experiments to a reference experiment in which the BB emissions were aged under UV lights (*via* the OH radical). The BB emissions in this experiment (experiment 9) were exposed to UV lights (*J*_NO_2__ of 0.59 min^−1^) for 3 h. The experiment under UV conditions had a larger increase in O : C ratio (0.32 or an 80% increase) than the experiments under dark conditions (0.09–0.23 or a 20–60% increase) after 3 h. Similarly, the UV experiment showed the largest change in theta angle (28° as opposed to 8–27° in the dark-aging experiments). Conversely, the SOA formation in the UV experiment tended to be similar or slightly lower than in experiments under dark conditions. In the UV experiment, the OA enhancement ratio was 1.47 (6 μg m^−3^), while in the dark oxidation experiments it ranged between 1.00–1.95 (0.1–74 μg m^−3^) with one experiment reaching an OA enhancement of 3.9 (experiment 6).

In experiment 10, where no external oxidation was initiated, the O : C ratio remained constant throughout the experiment and the theta angle between the bbOA at the end of the experiment compared to the emitted spectrum was only 1.3°, indicating near-identical spectra.

The fresh bbOA AMS mass spectrum exhibited moderate variability across the various experiments with an average theta angle between pairs of experiments of 17° (standard deviation of 7°, Fig. S1†). Among the dark oxidation experiments, the produced bbSOA spectrum tended to be more similar with (*i.e.*, a lower theta angle) between two experiments than the fresh bbOA factor for the same pair of experiments (an average theta angle of 12.5° with a standard deviation of 6°). This increase in similarity may be due to the increasing prominence of OA mass at *m*/*z* 28 (CO^+^), 29 (CHO^+^), and 44 (CO_2_^+^) in all aged bbOA spectra.

#### 3.2.2 Secondary inorganic and organic aerosol nitrate formation

The fresh BB particulate emissions included between 0.2–1.5 μg m^−3^ of total particulate nitrate (inorganic plus organic). We estimate that in all experiments, approximately 33–56% of the fresh BB PM nitrate was organic (Table 1). It is unclear if this variability was due to the properties of the wood fuel, combustion conditions, or temperature in these experiments.

**Fig. 5:**
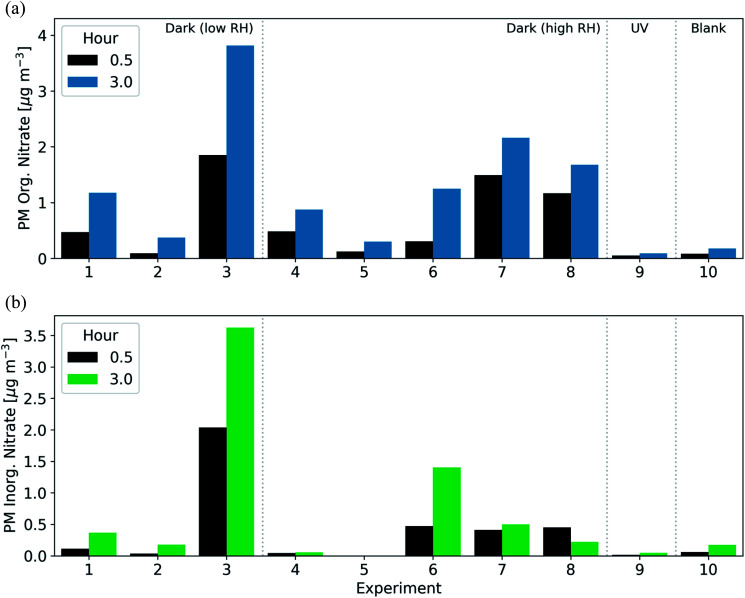
Production of secondary (a) PM organic nitrate and (b) PM inorganic nitrate aerosol after 0.5 h (black) and 3 h (blue/green) following the initiation of oxidation across all experiments.

After the injection of NO_2_ and O_3_, both organic and inorganic nitrate increased rapidly. In 7 of 8 experiments aged under dark conditions, the majority of the secondary nitrate aerosol was organic, resulting in 46–100% of the post-oxidation (after 3 h) nitrate aerosol being organic (Fig. 5). In the 30 min after the initiation of oxidation, the organic nitrate mass concentration increased by 0.1–1.9 μg m^−3^. For comparison, under UV conditions, the organic nitrate mass concentration increased by only 0.08 μg m^−3^ in the three hours after the initiation of oxidation (Fig. 5). The large enhancements of inorganic and organic nitrate aerosol are likely characteristic of dark oxidation due to the presence of NO_2_ and the NO_3_ radical.

### 3.3 Aerosol acidity

**Fig. 6:**
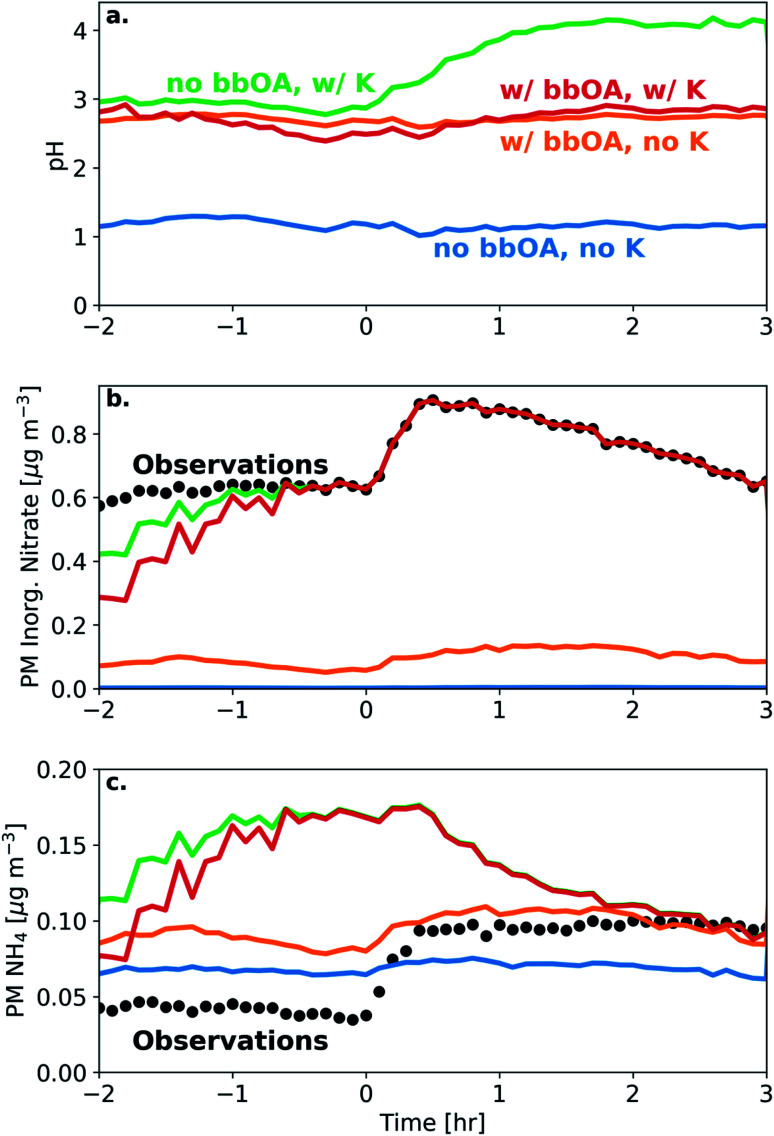
Model simulations and corresponding measurements for experiment 8 showing (a) aerosol pH, (b) PM inorganic nitrate, and (c) PM NH_4_. Included are sensitivity simulations with neither bbOA water uptake nor K included in the model (blue), only bbOA water uptake included (orange), only K included (green), and the base assumptions with bbOA water uptake and K included (red). Measurements from the HR-ToF-AMS are included as black dots.

We simulated the thermodynamic properties of the bbOA, in order to estimate the aerosol acidity before and after oxidation. Under the base assumptions (outlined in Section 2.4), we calculate an average pH for the fresh emissions of 2.8 (with standard deviation of 0.25) across all experiments (Table S1†). The pH of the aerosol did not change appreciably following dark oxidation, with an average pH of the aged aerosol of 2.8 (standard deviation of 0.12) (Fig. 6). The largest change in estimated pH occurred in experiment 6 with a 0.3 unit increase between the fresh (pH of 2.5) and aged (pH of 2.8) aerosol.

To test the sensitivity of including bbOA water uptake into the model and the assumed K^+^ levels, we perform additional simulations: (1) a simulation with no bbOA water uptake and K^+^ concentrations set to zero; (2) K^+^ concentrations equal to 15% of the PM nitrate (but without bbOA water uptake); (3) bbOA water uptake but with K^+^ concentrations set to zero; (4) and with both bbOA water uptake and potassium concentrations included in the model. When including neither bbOA water uptake nor K^+^ in the model, the pH of the BB aerosol stays roughly constant at a value of 1.2 throughout the experiment (Fig. 6). When bbOA water uptake alone is included, the pH of the bbOA increases to 2.7. Similarly, when an estimate of K^+^ emissions from BB is included (but without bbOA water uptake), the pH of the freshly emitted BB aerosol increases to 2.9. Interestingly, when K^+^ is included without bbOA water uptake, the pH of the BB aerosol increases from 2.9 to 4.1 following oxidation; however, when both bbOA water uptake and K^+^ are included the pH ranges from only 2.5–2.8 throughout the experiment.

These differences are due to the change in simulated PM inorganic nitrate and ammonium when bbOA water uptake and K^+^ are included in the model. When neither bbOA water uptake nor K^+^ are included in the simulation, nitrate is entirely in the gas phase (in the form of HNO_3_). Including bbOA water uptake leads to a slight increase in simulated particle-phase inorganic nitrate (0.1 μg m^−3^). However, when K^+^ is included, the simulated PM inorganic nitrate matches the observations after one hour. On the other hand, the inclusion of K^+^ leads to an over prediction of PM ammonium relative to the observations. This highlights the importance of both bbOA water uptake and K^+^ concentration in determining aerosol pH. This also means that while pH in biomass burning aerosol is buffered largely by the NH_3_–NH_4_^+^ partitioning,^71–73^ the amount of organic water uptake plays a pivotal role in the case of biomass burning to ensure that bbOA pH is virtually constant throughout its lifetime.

In additional sensitivity simulations, we tested a range of hygroscopicity parameters from 0.1 to 0.2, and found little change in estimated pH.

### 3.4 Equivalent atmospheric time scales

In a subset of experiments (experiments 1, 4, 5, 7, and 8), we measured the NO_3_ radical concentrations during the BB experiments. Prior to the initiation of oxidation, NO_3_ radical concentrations were below the detection limit (approximately 3 ppt for 10 s of acquisition time) of the instrument, suggesting that the NO_2_ and O_3_ emitted from this source were not sufficient to form substantial concentrations of NO_3_. In the 30 min following the injection of NO_2_ and O_3_, NO_3_ concentrations increased to 100–400 ppt for experiments 1, 7, and 8 (with relatively higher injections of NO_2_ and O_3_) and 0–50 ppt for experiments 4 and 5 (with relatively lower injections of NO_2_ and O_3_). In experiment 5, NO_3_ stayed near the detection limit of the instrument for the duration of the experiment. In the remaining four experiments (experiments 1, 4, 7 and 8) there were significant concentrations (80–400 ppt) of NO_3_ throughout the 3 h oxidation period, suggesting that our dark-aging experiments were likely limited by VOC levels as opposed to oxidants.

**Fig. 7:**
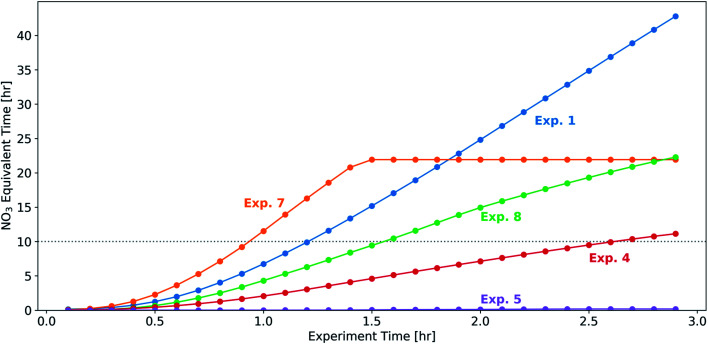
The equivalent number of hours in an ambient urban environment (assuming an NO_3_ radical concentration 20 ppt throughout the night) for the subset of experiments with NO_3_ radical measurements.

To relate the experimental timescale and injected concentrations of oxidant precursor species (NO_2_ and O_3_), we assume a typical nighttime ambient urban NO_3_ radical concentration of 20 ppt, in line with previous measurements at urban sites.^29,31,34,35^ In the 30 min following the injection of NO_2_ and O_3_, the NO_3_ exposure across the experiments with measured NO_3_ radical concentrations above the limit of detection (experiments 1, 4, 7, and 8) ranges from 0.4–2.3 equivalent hours. After 3 h, this range increases to 11–43 h. Assuming 10 h of darkness in an ambient environment, the integrated NO_3_ radical exposure after 0.9–2.8 h is roughly equivalent to one night of NO_3_ exposure in a typical urban environment (Fig. 7). As a comparison, we estimate OH exposure in the UV-aging experiment (experiment 9) and calculate that 3 h of experimental time is roughly equivalent to approximately 7–10 photochemical equivalent hours (assuming [OH] = 1.5 × 10^6^ molecule per cm^3^ throughout a 24 h period).

One important limitation in this analysis is the possibility of O_3_ or OH contributing to the oxidation in the dark-aging experiments. Following the discussion in Section 3.1, our estimates of the average lifetime of phenol, isoprene, and α-pinene suggest an important role of NO_3_ in the fate of all three VOCs (with average lifetimes one hour or less), while OH likely does not contribute to the oxidation of these VOCs (with the shortest estimated lifetime of six hours, or twice the length of the oxidation portion of the experiment; Table S4†). As discussed previously, O_3_ may contribute to reactions with α-pinene or to reactions with the products of the reaction of phenol with NO_3_. In this case, the equivalent time scale in an ambient urban environment for our experiments would be longer than discussed here, as the O_3_ concentration in our experiments (44–228 ppb) is higher than is typical in nighttime ambient environments. Further, this analysis does not take into account heterogeneous reactions which may affect the O : C ratio.

## 4 Conclusions and implications

In this study, we performed a suite of smog chamber experiments to explore the sensitivity of the chemical processing and formation of secondary aerosol of biomass burning emissions to aging under dark (dominated by the NO_3_ radical) conditions. The biomass combustion was designed to be representative of residential heating in an urban environment. We find that biomass burning organic aerosol (bbOA) does age rapidly under dark conditions following exposure to NO_2_ and O_3_. The oxygen-to-carbon ratio increased by 7–34% in the first 30 min following the injection of NO_2_ and O_3_. The experiments with the lowest increase in the oxygen-to-carbon ratio were experiments with the lowest levels of NO_2_ and O_3_ (suggesting low NO_3_ radical concentrations). Production of secondary organic aerosol across these experiments ranged from 0.7–38 μg m^−3^ (corresponding to a 4–81% increase over the initial emitted organic aerosol concentration). In addition, the dark-aging experiments produced 0.1–3.8 μg m^−3^ of secondary nitrate aerosol, much of which (46–100%) is in the form of organic nitrate. The results presented here are in broad agreement with the results of Tiitta *et al.*^46^ and Hartikainen *et al.*^47^ which reported large enhancements of secondary organic aerosol and consumption of phenolic compounds after exposure of biomass burning emissions to the NO_3_ radical.

In a subset of experiments, the NO_3_ radical concentration was measured using incoherent broad-band cavity-enhanced absorption spectroscopy (IBB-CEAS). In the 30 min following the injection of additional NO_2_ and O_3_, the NO_3_ radical concentration increased to as much as 400 ppt across these experiments. Assuming an average nighttime concentration of 20 ppt (typical of an urban environment), roughly 1–3 h of NO_3_ exposure at these experimental concentrations would correspond to one night of continued 20 ppt exposure (defined as 10 hours of darkness). While this comparison of NO_3_ radical exposure to ambient urban conditions is a rough estimate, it does suggest that freshly emitted bbOA can undergo substantial chemical processing, including the formation of secondary organic and inorganic aerosol, overnight.

We compare these dark-aging experiments to 2 reference experiments: one experiment with no external initiation of oxidation and one experiment with oxidation initiated with UV lights. While there were low levels of NO_2_ and O_3_ (7 and 23 ppb, respectively) emitted with the biomass burning OA and VOCs, the experiment with no external initiation of oxidation showed no increase in the oxygen-to-carbon ratio and only a slight increase in aerosol concentration (within the uncertainty range of the wall loss correction) after 3 hours of the experiment. During an experiment in which the BB emissions were aged under UV lights, the O : C increased more than in the dark-aging experiments (43% after 0.5 h and 81% after 3 h). Conversely the production of secondary organic aerosol was comparable to the dark-aging experiments; however, we note that we did not test the same range of initial conditions in the UV experiments as in the eight dark-aging experiments.

The enhancement in secondary inorganic and organic aerosol did not appear to substantially affect the calculated pH of the biomass burning aerosol if the water uptake from the organics is considered in the calculations. Using the ISORROPIA-Lite thermodynamical model, we estimate the pH of the BB aerosol is roughly constant at 2.8 before and after the dark oxidation. The modeled partitioning of ammonium is consistent with the observed values. This result does exhibit moderate sensitivity to the amount of potassium in the aerosol, which is largely mitigated from the presence of organic water. These levels of pH promote most of the total nitrate to be in the form of aerosol nitrate, which implies that its dry deposition rates in the ambient atmosphere would significantly decrease compared to if it were residing in the gas phase in the form of HNO_3_.^55^ This observation carries two significant implications: inorganic nitrate would tend to accumulate much more in the boundary layer before it deposits, increasing the PM levels considerably and its sensitivity to NH_3_ and/or HNO_3_,^54,55^ while the relatively slow deposition of nitrate means that the corresponding nitrogen can be transported over long distances before it is deposited.^54^ This in itself increases the range of influence of BB emissions, their impact on air quality and ecosystem productivity over large regions of the globe.^53^

There are a number of limitations regarding this study. First, we did not systematically test the sensitivity of the production of secondary aerosol under dark conditions to differences in combustion conditions. Different combustion conditions may alter the profile of gas- and particle-phase emission, and hence alter the degree to which the fresh emission may be aged by the NO_3_ radical. While our laboratory emissions are representative of residential heating, we note that open fires may behave differently. In addition, we note the possible loss of semi-volatile organic species during the transfer of the emission plume into the experimental chamber. Second, while the NO_3_ radical appears to dominate the oxidation process (the estimated average lifetime of phenol, isoprene, and α-pinene against reaction with NO_3_ are much less than the length of the oxidation portion of the experiment), it is possible that O_3_ may react with monoterpenes or with later-generation VOC products to enhance chemical aging. In this case, the equivalent time scales based on NO_3_ exposure would be an underestimate of nighttime oxidation in an ambient environment. Finally, when separating fresh from produced organic aerosol spectra, we assume only homogenous gas-phase oxidation takes place without heterogeneous reactions on the particle surface. Recent work by Yazdani *et al.* (*in review*) suggest that both aerosol- and gas-phase aging of primary BBOA may occur to a significant extent, perhaps more than AMS-based analysis alone may imply. Despite these limitations, our results suggest that biomass burning emissions are able to age substantially under dark conditions on a time scale of roughly one night under polluted urban conditions, and the inherent properties of the aerosol (acidity, hygroscopicity) favor the condensation of significant amounts of nitrate to the aerosol phase. This in turn reduces the deposition rate of nitrates, promotes the accumulation of nitrate aerosol in the boundary layer and its transport over long distances before deposition to the surface. Not including this dark aging mechanism and acidity effect in chemical-transport models underestimates the connection between biomass burning emissions and nocturnal air pollution.

## Data availability

Experimental results are available through the EUROCHAMP Data Centre (https://data.eurochamp.org/). The EUROCHAMP Data Centre is maintained by the French national center for Atmospheric data and services AERIS.

## Author contributions

J. K. K., A. N., and S. N. P. contributed to conceptualization; J. K. K., C. K., M. P., K. F., A. N., and C. V. contributed to investigation; S. J., M. C., M. C., A. N. and B. P. V., contributed to novel methods and instrumentation; J. K. K., C. K., M. P., K. F., S. J., A. N. and C. S. contributed to formal analysis; J. K. K. wrote the original draft; all authors contributed to review and editing.

## Conflicts of interest

There are no conflicts to declare.

## Supplementary Material

EA-002-D2EA00031H-s001
