# Supplementary material for: Secondary aerosol formation during the dark oxidation of residential biomass burning emissions
Source: Environ Sci Atmos. 2022 Aug 23;2(5):1221–36. doi: 10.1039/d2ea00031h (PMC9476557; doi:10.1039/d2ea00031h)
Supplement: EA-002-D2EA00031H-s001 [file EA-002-D2EA00031H-s001.pdf]

Electronic Supplementary Material (ESI) for Environmental Science: Atmospheres.
This journal is © The Royal Society of Chemistry 2022

Supplemental Material

Secondary aerosol formation during the dark oxidation of biomass burning emissions

John K. Kodros[1], Christos Kaltsonoudis[1], Marco Paglione[2], Kalliopi Florou[1], Spiro Jorga[3], Christina Vasilakopoulou[1,4], Manuela Cirtog[5], Mathieu Cazaunau[5], Bénédicte Picquet-Varrault[5], Athanasios Nenes[1,6], Spyros N. Pandis[1,4]

[1]Institute of Chemical Engineering Sciences, ICE-HT, Patras, 26504, Greece
[2]Institute of Atmospheric Sciences and Climate, Italian National Research Council, Bologna 40129, Italy
[3]Department of Chemical Engineering, Carnegie Mellon University, Pittsburgh, 15213, USA
[4]Department of Chemical Engineering, University of Patras, Patras 26504, Greece
[5]LISA, UMR CNRS 7583, Université Paris-Est Créteil, Université de Paris, Institut Pierre Simon Laplace (IPSL), Créteil, France
[6]School of Architecture, Civil and Environmental Engineering, Swiss Federal Institute of Technology Lausanne, Lausanne 1015, Switzerland

Table S1. Simulated pH using the base set of assumptions in a subset of experiments for fresh BB aerosol (defined as the average over hour -1 to hour 0) and aged pH (defined as the average over hour 2 to hour 3).

Experiment	Fresh pH	Aged pH
4	2.9	2.9
5	2.6	2.6
6	3.1	2.9
8	2.5	2.8

Table S2. Assumed reaction rate constants used to calculate typical lifetime of the VOCs with the largest observed decrease. Reaction rate constants are taken from the Master Chemical Mechanism, MCM v3.3.1 (Jenkin et al., 2003, 2015; Boss et al., 2005).

Oxidant	k_{phenol} (molecule^{-1} cm^{3} s^{-1})	$k_{isoprene}$ (molecule^{-1} cm^{3} s^{-1})	$k_{a\text{-}pinene}$ (molecule^{-1} cm^{3} s^{-1})	k_{C6H5O} (molecule^{-1} cm^{3} s^{-1})
NO_3	3.8 x 10^{-13}	7.0 x 10^{-13}	2-4 x 10^{-12}	-
O_3	-	2.5-3.8 x 10^{-18}	3.8-5.6 x 10^{-17}	2.9 x 10^{-13}
OH	1.7-3.9 x 10^{-12}	2.0 x 10^{-12}	3.9 x 10^{-12}	-
		2.9 x 10^{-11}	3.0 x 10^{-11}	

Table S3. Average concentrations of oxidants and estimated lifetimes for the VOCs with the largest observed decrease in Experiment 1.

Oxidant	Concentration (molecule cm^{-3})	τ_{phenol} (h)	$\tau_{isoprene}$ (h)	$\tau_{a\text{-}pinene}$ (h)	τ_{C6H5O} (h)
NO_3	8.1 x 10^{8}	0.9	0.5	0.08-0.16	-
O_3	2.6 x 10^{12}	-	28-42	1.9-2.9	4 x 10^{-4}
OH	9.5 x 10^{5}	74-173	10-146	9.7-74	-

Table S4. Average concentrations of oxidants and estimated lifetimes for the VOCs with the largest observed decrease in the subset of experiments where NO_3 is above the measured detection limit.

Oxidant	Concentration (molecule cm^{-3})	τ_{phenol} (h)	$\tau_{isoprene}$ (h)	$\tau_{a\text{-}pinene}$ (h)	τ_{C6H5O} (h)
NO_3	3-20 x 10^8	0.4-2.0	0.2-1.3	0.004-0.4	-
O_3	1-3 x 10^{12}	-	22-100	1.4-7	0.0003-0.001
OH	5-15 x 10^5	47-330	6-280	6-140	-

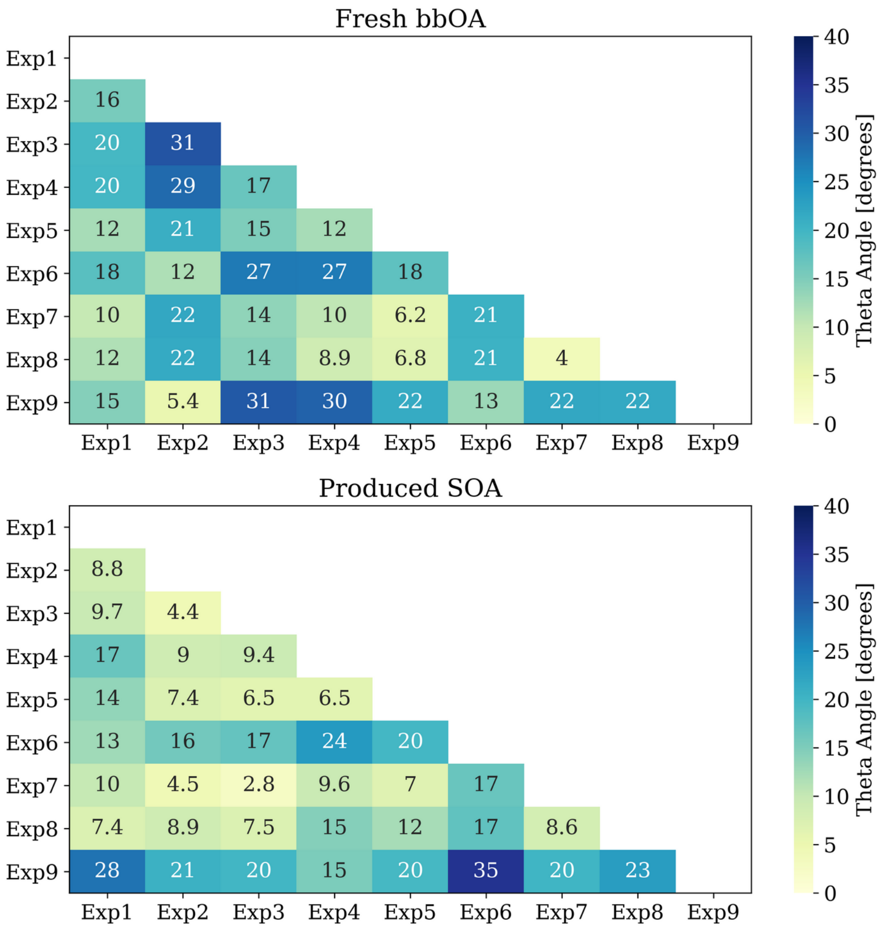

Figure S1. Comparison of theta angle across pairs of experiments for the OA mass spectra corresponding to a) fresh bbOA and b) produced bbSOA.

References

1. Bloss, C. *et al*. Development of a detailed chemical mechanism (MCMv3.1) for the atmospheric oxidation of aromatic hydrocarbons. *Atmos. Chem. Phys.* 5, 641–664 (2005).

2. Jenkin, M. E., Saunders, S. M., Wagner, V. & Pilling, M. J. Protocol for the development of the Master Chemical Mechanism, MCM v3 (Part B): tropospheric degradation of aromatic volatile organic compounds. *Atmos. Chem. Phys.* **3**, 181–193 (2003).

3. Jenkin, M. E., Young, J. C. & Rickard, A. R. The MCM v3.3.1 degradation scheme for isoprene. *Atmos. Chem. Phys.* **15**, 11433–11459 (2015).
